# Content-Specificity in Verbal Recall: A Randomized Controlled Study

**DOI:** 10.1371/journal.pone.0079528

**Published:** 2013-11-04

**Authors:** Jan Zirk-Sadowski, Denes Szucs, Joni Holmes

**Affiliations:** 1 Centre for Neuroscience in Education, University of Cambridge, Cambridge, United Kingdom; 2 Medical Research Council Cognition and Brain Sciences Unit, Cambridge, United Kingdom; University of Tokyo, Japan

## Abstract

In this controlled experiment we examined whether there are content effects in verbal short-term memory and working memory for verbal stimuli. Thirty-seven participants completed forward and backward digit and letter recall tasks, which were constructed to control for distance effects between stimuli. A maximum-likelihood mixed-effects logistic regression revealed main effects of direction of recall (forward vs backward) and content (digits vs letters). There was an interaction between type of recall and content, in which the recall of digits was superior to the recall of letters in verbal short-term memory but not in verbal working memory. These results demonstrate that the recall of information from verbal short-term memory is content-specific, whilst the recall of information from verbal working memory is content-general.

## Introduction

Working memory is the cognitive system responsible for the temporary maintenance and processing of information during complex cognitive activities. It is important for many everyday activities that require the online storage and processing of different types of information. These include reading comprehension, mental arithmetic, following directions, and reasoning [1–4]. In this experiment, we explore whether different types of verbal information (e.g. numbers and letters) are handled differently within different aspects of the verbal memory system.

There are several theoretical models of working memory which differ in their views of the nature, structure, and function of the system (see 5,6 for reviews). The primary distinction between these models is whether working memory is conceived of as a discrete entity (e.g., [7,8]) or a limited capacity process of controlled attention (e.g. [9–11]). 

One account, which is provided by the enduring model of Baddeley and Hitch [7,8] suggests working memory is comprised of four components. The central executive is responsible for monitoring and processing information across domains and for the retrieval of information from long-term memory and attentional control. Two storage systems, the phonological loop and the visuo-spatial sketchpad, provide temporary maintenance of verbal and visuo-spatial information. The fourth component, the episodic buffer, binds information across domains into integrated chunks [8]. 

Other accounts suggest that working memory capacity is limited by controlled attention that acts to activate existing representations in long-term memory in the face of distraction or interference [10,11]. In a latent factor analysis, Engle and colleagues [12] distinguished between verbal STM tasks and verbal working memory tasks, and herein lays the commonality across different models of working memory. Both accounts distinguish between the storage-only capacity of a verbal STM system and a central component that co-ordinates the ongoing processing of information with the storage of information in STM (see for example 7,12). This latter component, referred to as working memory, is more closely associated with measures of general intelligence, other higher order cognitive control functions and reading and mathematics ([9,10,13–15]). 

Verbal STM is a well-defined system, which is domain-specific and dedicated solely to storing verbal / phonological information. It is less clear whether working memory is domain-general (i.e. capable of manipulating and keeping active both verbal and visuo-spatial information) or whether there are separate subsystems for handling verbal and non-verbal information. Domain-general accounts of working memory capacity have been advanced by many leading theorists [10,16], and are supported by factor analytic studies in which tasks designed to measure the ability to process and store verbal and visuo-spatial information load on to a common factor (e.g. [17,18]). An alternative account is that working memory capacity is supported by two separate pools of domain-specific resources for verbal and visuospatial information ([19], see also 20). According to this account, each domain is independently capable of manipulating and keeping information active. Research on adult participants and on older children supports this distinction ([21–23]). 

Based on studies that have explored the contribution of short-term and working memory performance to other higher-level abilities such as reading and maths (e.g. [24]), our view is that working memory has a multi-component structure that includes a domain-general processing component (akin to Baddeley’s central executive) and domain-specific storage components (verbal STM/phonological loop and visuo-spatial sketchpad), and that working memory can be assessed by either verbal or non-verbal tasks that involve both the storage and manipulation of information. In this study, we use a verbal working memory task to facilitate a comparison of the handling of phonological information in verbal STM and verbal working memory, but we believe that verbal and visuo-spatial working memory may have a common central executive component.

Although the distinction between verbal STM and working memory is relatively well understood, less is known about how different types of auditory/verbal material (e.g. digits or letters) are processed within the two systems. If working memory for verbal stimuli operates in a less specific way than verbal STM, we might expect differences in the way in which different forms of verbal material are handled across the two systems. If, as we suspect, working memory for verbal stimuli is more content-general than verbal short-term memory, there will be no differences in the recall of different forms of verbal information in verbal working memory but there will be in verbal STM. Thus, if there is an interactive effect of stimuli type and verbal memory component, we will establish further evidence for a distinction between verbal STM and verbal working memory by demonstrating differences in the way they handle different types of phonological information. 

Thus, the principal aim of the current experiment was to investigate whether there are content-specific effects within verbal STM and verbal working memory using carefully designed digit and letter stimuli. Although a small number of studies have previously compared memory for different verbal materials within STM or working memory ([25,26]), no single study has directly compared recall for different phonological materials across the different systems. Working memory is typically measured by tasks that involve the concurrent storage of information whilst processing additional, sometimes unconnected, information. STM tasks do not involve processing, and therefore typically require the immediate serial recall of information 2 tasks that are widely used in the literature to distinguish short-term and working memory, and which are used in the current study, are forward and backward span (e.g. [12,17]). Here we compare participants’ verbal recall in forward and reverse serial order using verbal stimuli that control for the potential confound of the mental representation of numerical and non-numerical ordinal sequences: the distance effect. 

The numerical distance effect describes how the ability to discriminate between two numbers improves as the numerical distance between them increases (e.g. [27–29]). So, for example, it is easier and faster to discriminate between ‘5’ and ‘9’ compared to ‘5’ and ‘6’. The same psychophysical *distance effect* has also been observed for letters ([30–33]). Based on these observed distance effects, it may be reasonable to assume that estimates of span, and importantly differences in performance with different modalities (e.g. digits or letters), could be confounded by distance effects if strings of letters or digits are presented with varying distances between stimuli (e.g. “8, 1” in a digit recall task might be more difficult to process than “A, M” in a letter span task where the distance is larger). In the current study, we use digit and letter span tasks that are matched for inter-stimuli distances across modalities to exclude this potential confound.

We predict a main effect of content, but our hypothesis is not directional. We consider both possibilities: i) performance on digits may be significantly better compared to letters (e.g. daily life circumstances require remembering digit strings such as telephone numbers, dates, postcodes, etc; also digits have a semantic sense while letters in isolation have no semantic loading; people easier remember meaningful stimuli); ii) performance on letters may be significantly better compared to digits (e.g. people practice recalling letter strings when learning the alphabet, the spelling of new words or when applying ordinal labels to objects: e.g. a, b, c, etc;). We also predict superior recall for forward span tasks based on the additional processing load associated with recalling information in reverse order in the backward span tasks ([34,35]). No a priori predictions are made about the interaction between content (letters and digits) and verbal memory type (forward recall, STM and backward recall, working memory).

## Materials and Methods

### Ethics Statement

This study received ethical approval from the University of Cambridge Psychology Research Ethics Committee. All participants gave informed written consent prior to participating. The full dataset is available upon request.

### Participants

Thirty-seven postgraduate students from the University of Cambridge participated in the experiment (24 females, age: 25.19 years (SD=2.55; Range = [20.59, 30.72]). All participants were native English speakers. Participants were paid for their time. 

### Measures

Participants completed the forward (FD) and backward (BD) Digit Span subtests of the Wechsler Adult Intelligence Scale, 3rd Edition (WAIS-III, [36]) to measure verbal STM and working memory respectively. In both tasks, sequences of digits were presented auditorially for immediate verbal recall in either forward serial order (FD) or reverse order (BD). Each task began with two trials at a sequence length of two items. Sequences increased by one digit every two trials, up to a sequence (span length) of 9 items. In total, there were eight blocks of trials, with two trials in each block (totalling 16 trials per task). Both tasks were administered according to the instructions of the WAIS-III manual ([36]). The experimenter read the sequences of letters/digits to the participant at a rate of 1 item per second. Each trial was scored as correct (1) or incorrect (0), as per the test manual. 

Parallel forward (FL) and backward (BL) letter span tasks were constructed by matching letters to each of the digits in the FD and BD tasks (e.g. 1 corresponded to A, 2 to B, 3 to C and so on). The distance between the letters was matched to the distance between the numbers in each of the trials in the Digit Span tasks. For example, if the first trial in FD was “1,3”, the corresponding FL trial was constructed as “A,C”. Task administration was identical to that of the Digit Span tasks. 

The same researcher administered all tasks to all participants. The tasks were administered in a fully randomized order, thus the study had a randomized controlled within-subjects design. 

## Results

Descriptive statistics summarising performance in each of the conditions are shown in Table 1. 

**Table 1:** Trial correct across the verbal memory tasks.

	Mean	SD	Min.	Max.
Digits forward	12.86	1.84	9	16
Letters forward	10.78	1.58	8	13
Digits backward	9.95	2.66	3	14
Letters backward	8.84	2.57	4	14
Average digit recall (forward and backward recall)	11.41	1.91	8	14.5
Average letter recall (forward and backward recall)	9.81	1.78	7	13.5
Average backward recall (letters and digits)	9.39	2.27	3.5	14
Average forward recall (letters and digits)	11.82	1.48	9	14.5

Table 2 shows the correlations between the tasks. There were significant associations between the two forward recall tasks (*p*=.002) and the two backward recall tasks (*p*=.001), consistent with measures of STM and working memory. The two letter recall tasks were significantly related to one another (*p*=.005), but there was not a significant association between the letter span forward and digit span backward tasks (*p*=.358). 

**Table 2:** Pearson correlations between measures.

	Digits forward	Letters forward	Digits backward	Letters backward
Digits forward		0.49**	0.43**	0.45**
Letters forward			0.16	0.45**
Digits backward				0.52**

A maximum-likelihood mixed-effects logistic regression [37–39] was conducted to test whether content (digit vs. letter) and memory component (STM, forwards vs. working memory, backwards) predict performance. This analytic approach for repeated measures data is more powerful than ANOVA [40]. The dependent variable entered into the regression model was memory performance, with memory type (STM or working memory, coded as 0 and 1 respectively) and content (digit, 0; letter, 1) entered as independent variables. Both were significant (content: digit vs. letter, β=-.461, p<.001, and memory type: forwards vs. backwards, β=-697, *p*<.001). The odds of correct performance were greater for digits than letters (odds ratio: OR=.498) and greater for forward than backward recall (OR=.631), indicating main effects of both content and memory type.

**Figure 1:**
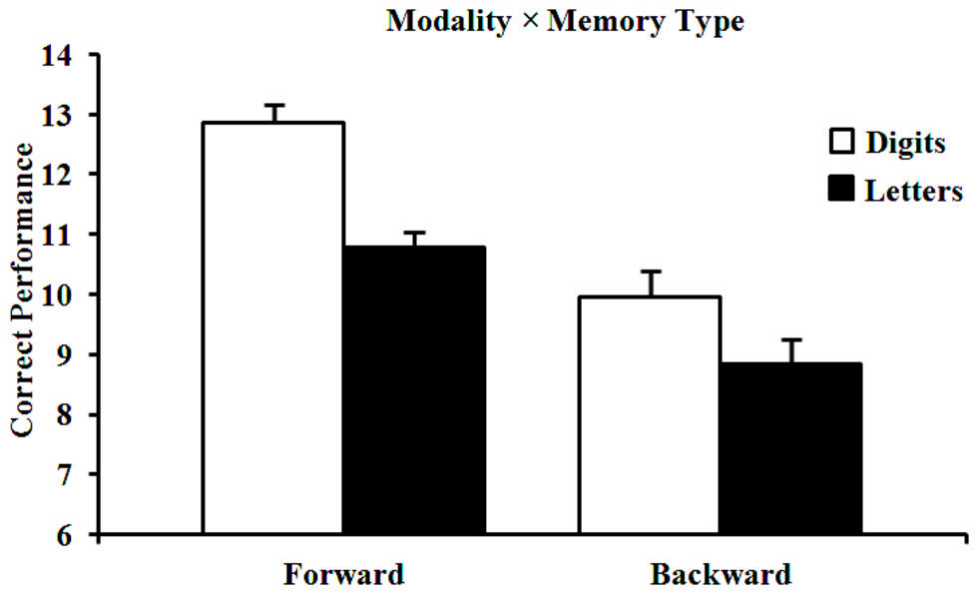
Content effects in forward (STM) and backward recall tasks (working memory). Error bars are standard errors of the mean.

The interaction term (content × memory) was entered into the model to test whether content effects were specific to one of the components of working memory. The 2 main effects remained significant (content, β=-.686, p<.001 and memory type, β=-.915, *p*<.001; with odds ratios of OR=.504 and OR=.400, respectively). The interaction between content and memory-type predicted performance, β=.399, *p*=.003 (odds ratio =1.491), with markedly better recall of digits compared to letters in forward recall (STM) (see Figure 1). 

## Discussion

The purpose of the present study was to investigate whether different types of information (digits and letters) are handled differently in verbal STM and verbal working memory. The results replicate the well-established distinction between STM and working memory and provide novel data demonstrating that the recall of information from verbal STM is content-specific, whilst the processing of information in working memory for verbal stimuli is content-general.

 By comparing performance on forward and backward digit recall tasks, we have demonstrated that participants are better able to recall information in forward than reverse order. Finding a main effect of recall (forward or backward) supports a distinction between STM and working memory (e.g. [10,11,41–43]), here probed in the context of phonological domain. It also provides further validation for the use of backward recall tasks as measures of verbal working memory (e.g. [44]), rather than STM (e.g. [10]). 

The second main effect, that the recall of digits is superior to the recall of letters, is as predicted and lends support to the notion that the extensive practice of remembering digits in everyday life (e.g. dates and telephone numbers) may facilitate performance over remembering arbitrary strings of letters. However, the superior recall for digits over letters was specific to only one aspect of the verbal memory system. Content-specific effects do provide a comprehensive account of the data in the short-term memory. This may be related to the lower activity of the central executive module in this context. Alternatively, numerical semantics may be processed more centrally in the forward recall. Semantic characteristics may be processed only peripherally in the working memory context: participants’ cognitive resources or attention may be more intensively spent on information retrieval rather than semantic processing in the backward recall tasks. The forward recall may facilitate semantic processing; hence larger difference between digits and letters.

A significant interaction was observed between content-type and memory, indicating that the verbal recall of different types of information differs between verbal STM and verbal working memory. Whilst participants were better able to recall digits than letters in forward order, this effect was not observed for backward order. This suggests that verbal STM is content-specific and (verbal) working memory is content-general. In terms of the theoretical structure of working memory, this is consistent with the view that the storage aspects of the system are more highly specialized and defined than the domain-general central executive system (e.g. [7]). According to multiple models there are distinct components associated with the storage of verbal and visual material (e.g. the phonological loop / verbal short-term memory and the visuo-spatial sketchpad, [7,11,17,24]). The current data go one step further to imply there may be separate systems, or at least distinct processes, associated with the storage of different forms of information within each of these storage-only systems. In terms of the working memory, or central executive system, the absence of a content effect provides further support for the notion of a domain-free processing ability that deals with all types of information, whether it is verbal or visuo-spatial (e.g. [24]), numerical or letter-based. Of course, further research is needed to investigate this, which could be achieved through investigating whether other types of phonological information (e.g. words and nonwords) interactively predict verbal STM and verbal working memory capacity, or whether there are similar content-specific effects in visuo-spatial STM using visuo-spatial working memory tasks. 

The current findings are important for the assessment of verbal STM skills. Because the content of to-be-remembered information influences performance, it is important that assessors give careful consideration to the type of material / stimuli presented to avoid over- or under-estimating ability. This is particularly important when assessing for potential deficits in disorders such as dyslexia where the immediate recall of phonological information is used as a marker of impairment. To ensure a fair estimate of performance is obtained, it would be advisable to consider using multiple assessments that cross different content-types. 

In summary, the findings from the current study indicate that forward and backward span tasks assess different aspects of the verbal memory system and that verbal STM recall is content-specific whilst working memory related verbal recall is content-general. 
